# Dilemmas concerning heart procurement in controlled donation after circulatory death

**DOI:** 10.3389/fcvm.2023.1225543

**Published:** 2023-07-31

**Authors:** S. E. Kaffka genaamd Dengler, M. T. Vervoorn, M. Brouwer, J. de Jonge, N. P. van der Kaaij

**Affiliations:** ^1^ Department of Cardiothoracic Surgery, University Medical Center Utrecht, Utrecht, Netherlands; ^2^ Department of Surgery, Erasmus Medical Center Transplant Institute, Rotterdam, Netherlands

**Keywords:** ethics, controlled donation after circulatory death heart transplantation, thoracoabdominal normothermic regional perfusion, determination of death, ex-situ heart perfusion

## Abstract

With an expanding population at risk for heart failure and the resulting increase in patients admitted to the waiting list for heart transplantation, the demand of viable organs exceeds the supply of suitable donor hearts. Use of hearts after circulatory death has reduced this deficit. Two primary techniques for heart procurement in circulatory death donors have been described: direct procurement and perfusion and thoraco-abdominal normothermic regional perfusion. While the former has been accepted as an option for heart procurement in circulatory death donors, the latter technique has raised some ethical questions in relation to the dead donor rule. In this paper we discuss the current dilemmas regarding these heart procurement protocols in circulatory death donors.

## Introduction

Since the criteria for determination of brain death were introduced in 1968 (1), heart transplantation relied on donor hearts from donation after brain death (DBD). Use of hearts from controlled donation after circulatory death (cDCD), where death is defined by the irreversible cessation of circulation (2), has proven to increase the availability of donor hearts of acceptable quality (3–7). Due to the expanding population at risk for heart failure and the resulting increase in patients admitted to waiting lists for heart transplantation, the current demand for viable organs exceeds the supply of suitable donor hearts, resulting in increased waiting list mortality (8) and strict criteria for waiting list admittance. Expanding the donor pool with cDCD donors can help to reduce this deficit (9).

For heart procurement in cDCD, two principal techniques have been described: direct procurement and perfusion (DPP) and procurement after thoraco-abdominal normothermic regional perfusion (TA-NRP), where extracorporeal membrane oxygenation (ECMO) is started in the donor after declaration of death (3–7). While the former has been widely accepted as an option for heart procurement during cDCD, the latter technique is not universally applied and has raised ethical questions despite the consensus document of the International Society for Heart and Lung Transplantation that states that TA-NRP is ethically justifiable (10). This ongoing debate over TA-NRP seems to originate from different interpretations of the concept of circulatory death, the interpretation of mechanical resumption of circulation in light of the dead donor rule (DDR), and the differences in length of the “no-touch*”* period (11). For instance, some approach circulatory cessation in the setting of cDCD as the defining moment at which the process of brain mortification ensues, as brain perfusion is absent from that moment onwards (12–15). This implies that as long as the process of brain mortification is not disturbed, mechanical resumption of circulation is in accordance with circulatory death and places emphasis on the central role of the brain in distinguishing life from death. On the other hand, others postulate that circulatory death in cDCD is based on cessation of circulation in itself, and for that reason restoration of any form of circulation (including mechanical) should not be attempted (16, 17). Similarly, differences in criteria that define circulatory death exist, with pulselessness (mechanical asystole) or absent electrical activity (electrical asystole) as different markers of cessation of circulation. And last, differences in the “no-touch” period, which is meant to ensure the absence of spontaneous return of circulation and ranges from 2 to 20 min, are also present (11).

In this review we provide an overview of the different points of view regarding cDCD, how it relates to the DDR and also discuss the ethics surrounding different procurement techniques (DPP vs. TA-NRP). In order to do so, some general concepts are discussed prior to providing ethical considerations. The goal is to summarize this topic for the reader, in order to facilitate consensus formation regarding the application of TA-NRP vs. DPP and highlight why this is such a precarious topic.

## Organ donation and the dead donor rule

In 1999, Robertson et al. formulated the principles of the DDR. The DDR was formulated in order to dissociate organ procurement from the act of killing in order to protect the patient from harm and preserve public trust in organ donation (18). In its current form, the DDR is twofold. First, the organ retrieval itself should not be the cause of death of an individual that is willing to donate. Second, death itself must be declared prior to organ procurement. The DDR is currently widely embedded within laws and regulations regarding organ and tissue donation and can be perceived as the ethical foundation on which the process of organ donation is built (19–22).

## Donation after circulatory death

Donation after Circulatory Death, previously described as non-heart-beating donation, was adopted during the early years of organ transplantation, but was abandoned after the introduction of the formal brain-death criteria in 1968. It was, however, reinstated after the first International Workshop on this topic in 1995. The classification that originates from this meeting (Maastricht Classification), describes the different types of donors according to the cause of cessation of circulation. It distinguishes controlled from uncontrolled cessation and thereby allows physicians to distinguish more suitable from less suitable potential donors (23). The last 25 years, this classification has been updated multiple times (24), with the last changes being made in 2013 during the DCD conference in Paris (Table 1) (25).

**Table 1:** Classification of circulatory death donors (24, 25).

**Category I** **Uncontrolled**	**Found dead**	Sudden unexpected circulatory arrest without any attempt of resuscitation by medical team
	IA. Out-of-hospital	
	IB. In-hospital	
**Category II** **Uncontrolled**	**Unsuccessful resuscitation**	Sudden unexpected irreversible circulatory arrest with unsuccessful resuscitation
	IIA. Out-of-hospital	
	IIB. In-hospital	
**Category III** **Controlled**	**Withdrawal of life-sustaining therapy**	Planned withdrawal of life-support with anticipated circulatory arrest
**Category IV** **Uncontrolled controlled**	**Circulatory arrest while brain dead**	Sudden circulatory arrest after brain death diagnosis during donor life-management but prior to organ recovery
**Category V** **Controlled**	**Euthanasia**	Organ donation after euthanasia

In short, the Maastricht classification makes a distinction between (1) a patient that has died outside of the hospital; (2) a patient that has died after unsuccessful resuscitation; (3) a patient that is withdrawn from life-sustaining treatment, but does not meet brain-death criteria; (4) the patient meets brain-death criteria and has a spontaneous circulatory arrest; and (5) the patient has died after euthanasia. According to this classification, only categories 3 and 5 are classified as cDCD.

## Defining circulatory death

Historically, the heart is often perceived as the primary driving force of life. For this reason, the presence of a heartbeat distinguished the living from the dead. Over the course of history, efforts were made to record specific signs to properly determine death and since the late 1800s, death is determined based on three vital signs: the absence of heartbeat, respiration and neurological function (26). These three vital signs also constitute important criteria for the determination of circulatory death, which is described as the irreversible cessation of circulatory and respiratory function, which marks the start of the process of brain mortification and irreversible loss of brain function (2, 20).

Although the concept of loss of circulation and respiration seems clear, a difference in criteria used to define loss of circulation is present across countries. For instance, some use equilibration of arterial/venous pressures (mechanical asystole), while others prefer electrical standstill (electrical asystole) (4, 27, 28). These differences are also reflected in guidelines and statements (29). Although electrical standstill is still used, detecting electrical activity is not by definition an indication of contraction or circulation.

## Duration of the “no-touch” period

To ascertain this irreversible loss of spontaneous circulation and respiration in the context of cDCD, all countries maintain a “no-touch” period (Table 2). The “no-touch” period represents the time between cessation of circulation (mechanical or electrical asystole) and the moment death is formally declared, in order to exclude the possibility of autoresuscitation. As the name implies, the process of dying may not be disturbed or influenced during this period. The exact duration of the “no-touch” period varies between counties (Table 2), ranging from 2 to 20 min. In general, 2-5 min of absent circulation are used to confirm irreversible loss of circulation, respiration and brain function, although discussion remains whether 2 min is an adequate length to exclude the possibility of autoresuscitation (30, 33). An European working group agreed on 5 min of absent circulation during the 6th International Conference in Organ Donation held in Paris in 2013 (34). A recent study, which investigated the resumption of cardiac activity after withdrawal of life-support in 631 patients, showed that no resumption of cardiac activity was seen after 4 min and 20 s (35). In our experience, 5 min of “no-touch” is sufficient to ascertain permanent cessation of circulation without the possibility of autoresuscitation if the left atrium is incised before the start of ventilation to prevent restart of cardiac perfusion by ventilation. The International Society for Heart and Lung transplantation has endorsed 5 min “no-touch” period (10). In our opinion, the “no-touch” period should be universally set at 5 min to eliminate existing differences and to prevent autoresuscitation.

**Table 2:** “No-touch” period per country (11, 30–32).

Country	“No-touch” period (minutes)
*Australia*	2–5
*Austria*	10
*Belgium*	5
*Canada*	5
*Czech Republic*	5
*France*	5
*Ireland*	10
*Israel*	5
*Italy*	20
*Latvia*	5
*Lithuania*	5
*Netherlands*	5
*Norway*	5
*Poland*	5
*Portugal*	10
*Russia*	30
*Spain*	5
*Sweden*	5
*Switzerland*	5
*United Kingdom*	5
*United States*	2–5

## Current methods for heart procurement in circulatory death donors

Different techniques have been developed for the procurement and preservation of cDCD hearts: direct procurement followed by static cold storage (DP-SCS), direct procurement followed by ex-situ heart perfusion (DP-ESHP), procurement after thoraco-abdominal normothermic regional perfusion in the donor followed by ex-situ heart perfusion (TA-NRP-ESHP) and procurement after thoraco-abdominal normothermic regional perfusion in the donor followed by static cold storage (TA-NRP-SCS) (36, 37).

In case of ESHP, the heart is mounted on a device for hypothermic or (sub)normothermic machine perfusion and preserved in an unloaded state. In case of TA-NRP, ECMO is initiated in the donor after declaration of death, while perfusion of the brain is prevented by occlusion of the aortic arch vessels. As a result, the heart resumes its contractile function due to the re-establishment of coronary perfusion while the brain is thought to be devoid of circulation. After a recovery period, the donor may be weaned from ECMO, and the cardiac contractile function can be evaluated in-situ prior to organ procurement, ensuring adequate graft quality after the inevitable warm ischemic insult associated with circulatory death. Regarding the clinical application of cDCD with the purpose of heart transplantation, DP-SCS has been found to be futile due to inadequate quality of the harvested hearts, since SCS will not restore the ATP levels in the heart. The first success with the DP-ESHP technique was reported in 2015 (3), which was quickly followed by TA-NRP-ESHP (4) and TA-NRP-SCS cases (38) Currently, six countries have a cDCD heart transplantation program (Table 3).

**Table 3:** Type of heart procurement in circulatory death donors per country (11).

Country	Type of procurement
*Australia*	DPP
*Belgium*	DPP/NRP^a^
*Canada*	DPP^b^
*Netherlands*	DPP
*United Kingdom*	DPP/NRP
*United States*	DPP/NRP

^a^ Currently halted.

^b^ Under governmental review (12).

## Benefits of TA-NRP

The most important benefit of TA-NRP is that it enables quality assessment of the heart after the warm ischemic insult compared to DP-ESHP. TA-NRP therefore facilitates better selection of donor hearts (5). Improved selection may result in a lower complication rate post-transplantation and better survival. Physiologically, TA-NRP has the benefit that the kidneys and the liver provide the optimal homeostatic environment compared to the recirculating diluted whole blood in the DP-ESHP setting which could impact endothelial and thus graft function of multiple organs. Until now, no data is available that shows superiority for TA-NRP in cDCD heart transplantation (5) but, although numbers are small, multiple studies show 100% survival at 30 days with TA-NRP (5, 39–41). This data suggest that in a larger study, the advantage of TA-NRP may be demonstrated if the survival gap between the groups persist. Also, TA-NRP allows multiple organs to be perfused at once, which could reduce the need for multiple perfusion machines. It improves the quality of kidneys and livers and has shown superior outcomes after transplantation for kidneys and livers compared to other preservation techniques (42–46). Furthermore, TA-NRP gives the opportunity to preserve hearts using SCS after TA-NRP, analogous to what is done with brain death donation (38). The use of SCS instead of ESHP after TA-NRP will directly lead to a reduction in healthcare costs (4–6), by saving disposables ($50.000) and human resources needed for DP-ESHP. The question therefore arises whether TA-NRP should also become the golden standard of procurement in a cDCD setting, following the example set by centers in the United Kingdom and Belgium. However, re-establishment of circulation by restoring thoraco-abdominal perfusion in a donor that has been declared dead on circulatory grounds, raises ethical concerns not only because of the possibility of brain perfusion. This is the reason why TA-NRP is currently infrequently performed in the United Kingdom and Belgium.

## The ethical dilemmas regarding TA-NRP

When assessing the literature, most of the ethical dilemmas surrounding TA-NRP seem to originate from the interplay between the DDR and the formal declaration of death. Although a formal definition of circulatory death is available, it remains subject to interpretation and (local) beliefs, especially regarding what distinguishes life from death in this sense. Below we highlight some important aspects of these ethical dilemmas.

### Defining circulatory death

Most laws and regulations use the term irreversible cessation of circulation and respiration in order for death to be declared on circulatory grounds. Irreversible is referring to a condition that cannot be restored no matter what, yet both can be mechanically resumed with life-sustaining therapies. Alternatively, one could use “*permanent*” cessation instead of “*irreversible*” (47–49). Whereas irreversible cessation means that the function *cannot* resume, permanent cessation means that it *will* not resume and is thus dependent on action and intent (47, 48). This means that cessation is *permanent* but not necessarily *irreversible*.

Although death is determined differently for circulatory death and brain death, they are essentially based on the same principle: the cessation of the ability for spontaneous, integrated functioning of multiple bodily systems, with human consciousness as the best example (26). Fundamentally, this integrated functioning is dependent on oxygen delivery to the brain, which orchestrates consciousness (cortex) and spontaneous bodily functions, such as breathing and blood pressure regulation (brain stem). Based on this principle, some have argued that in the setting of circulatory arrest, death can be determined based on permanent cessation of brain circulation and subsequent irreversible loss of brain function (26, 47, 49). This approach places emphasis on the central role of the brain in distinguishing life from death. Taking this into account, this approach suggests that mechanical restoration of circulation (TA-NRP) to other organs than the brain would be feasible as long as restoration of blood flow to the brain would be prevented (13, 14, 47, 50). This is the basis on which TA-NRP is introduced in the United Kingdom, Belgium and in the United States (39, 51).

On the other hand, if emphasis is placed on permanent cessation of circulation and respiration as defining features of circulatory death, this means that no mechanical circulation in-situ should be initiated. This is currently the case in Australia, where cDCD heart transplantation is performed using DP-ESHP only (50) and this approach excludes the use of TA-NRP altogether.

To summarize, the application of TA-NRP depends partly on how circulatory death is defined in the cDCD setting and to what extent emphasis is placed on the functioning of the brain vs. the presence of (mechanical or spontaneous) circulation itself.

### The “no-touch” period and irreversible loss of brain function

As described previously, the “no-touch” period represents the time between cessation of circulation and the formal declaration of death and its main purpose is to exclude the possibility of autoresuscitation. Yet, if we choose the absence of brain perfusion as the final denominator of circulatory death, how would that impact the “no-touch” period to ensure irreversible loss of brain function after cessation of brain perfusion? Little evidence exists on the time when irreversible loss of brain function occurs. One review describes that in the clinical setting, loss of consciousness and loss of spontaneous electroencephalography activity occurs within 30 s after circulatory arrest (52). However, evoked cortical activity may persist up to several minutes. This might be even longer for brainstem evoked potentials, as this area of the brain is more resilient to anoxia-ischemia (52). Furthermore, the absence of spontaneous electrical activity is accepted as a marker of absent consciousness and awareness, although it cannot guarantee it (53).

Therefore, if emphasis is placed on the central role of the brain in distinguishing life from death to justify TA-NRP with exclusion of the cerebral circulation to avoid disruption of the process of brain mortification, we should be aware of the exact moment that irreversible cessation of brain function is reached before initiating TA-NRP, or be absolutely sure that execution of TA-NRP with exclusion of the cerebral circulation does not disturb the process of brain mortification. In the first case, this might warrant adaptation of the “no-touch” period to exclude any possibility of persistent brain function, instead of excluding the possibility of autoresuscitation. In the second case, sufficient evidence is needed to ascertain that TA-NRP with exclusion of the cerebral circulation will not result in restoration of brain perfusion and harm the process of brain mortification. Recent studies demonstrate no signs of brain perfusion and function after clamping the aortic arch vessels, compared to a control group that showed (partial) resumption of brain perfusion and activity (54). Other studies support this (55–57). In case of doubt or concern regarding potential collateral circulation to the brain, draining the arch vessels to atmospheric or negative pressure might divert any collateral flow away from the brain and may be an alternative to clamping these vessels (50). Based on these findings, one could state that TA-NRP with clamping or drainage of the aortic arch vessels does not restore brain perfusion and function, and hence does not harm the principle of permanent cessation of brain function required to declare a patient dead on the basis of circulatory grounds with the brain as final denominator of circulatory death. Yet, the number of patients in these studies is low and anatomic variation in collateral circulation to the brain might not have been investigated in these studies.

### Resuscitation or donor treatment?

Another important factor to address is whether TA-NRP is considered as resuscitation itself, or donor treatment in line with the necessary investigations before acceptance of donor organs for transplantation. On the one hand, initiation TA-NRP can be viewed as resuscitation if emphasis is placed on the absence of circulation itself as the final denominator of circulatory death. After all, an attempt is made to undo the situation that is responsible for the death of the donor and can thus be seen as resuscitation. In this approach, the donor is only dead as long as circulation is absent and restoration of circulation is thus viewed as harming the criteria on which circulatory death is defined, regardless of neurological status. Furthermore, the act of active exclusion of the cerebral circulation during TA-NRP could also be viewed as an intervention aimed at preventing restoration (i.e., resuscitation) of brain function and thus inflicting damage to the patient, which is now considered alive on the basis of presence of (mechanical) circulation, and could thus be viewed as inflicting brain death in an otherwise alive (on circulatory grounds) patient. In this case, the DDR is not respected since the donor is considered not dead at the time of organ procurement.

On the other hand, if the decision has been made to withdraw life-sustaining therapy based on an unfavorable prognosis, the sequential nature of observing cessation of spontaneous circulation and breathing, combined with the independent and deliberate decision to not attempt resuscitation and maintain the appropriate “no touch” period, death itself has been declared prior to organ procurement. The definition of circulatory death is met because the deliberate decision has been made not to resuscitate the patient in their best interest (34, 47). The fact that TA-NRP only results in “mechanical” restoration instead of autoresuscitation, fits this narrative, especially in case the cerebral circulation is actively excluded and the process of brain mortification is not disturbed (34). In this line of reasoning, the act could be seen as part of the necessary investigation before acceptance of the donor organs for transplantation.

### Societal perspective

Since most organ donation systems rely on confidence of the community in donation on voluntary grounds, we should not forget the impact on society. The medical world can postulate evidence-based arguments, but culture, emotions, habits and beliefs will play a role in how society will weigh circulatory death and TA-NRP. Whereas medical professionals tend to view death as a process with a clear starting point based on physiologic parameters, others might view death as a process that should not be disturbed. And if we agree on the brain as final denominator of circulatory death, to some, the beating heart is still regarded as a sacred organ that possesses special value of life. Caution with the introduction of TA-NRP is therefore necessary, as it could harm the perceived safety and the protection of the individual human within society, as death, and thus donation, might be experienced differently. Since donation is a voluntary act, it is important to maintain societal confidence in systems that guide this. Losing confidence would be very damaging and undo most of the progress we have made in the past, thereby defeating its own purpose.

### Alternative to TA-NRP—abandoning the DDR

Abandoning the DDR would constitute a paradigm shift in the ethics of deceased organ procurement from donor beneficence to donor autonomy and non-harmfulness (58, 59). This switch would require changes in laws to legitimize organ procurement during the dying phase of the donor and public discussion about autonomy-based end-of-life decisions. Practically, euthanasia through organ donation could be an option in which voluntary informed consent is key. In this regard, Verheijde described the option of organ procurement after voluntary informed consent as mentioned by Truog (59, 60); the preservation of a person's autonomy and the voluntary nature of the decisions is fundamental for organ procurement in the dying phase. Mandated choice, in which an individual is required to document their decision, is thought to guarantee autonomy and the voluntary nature, although opponents may postulate that it is unacceptable in a libertarian society (59). Bollen explored anesthetized organ procurement and concluded that the consequentialist and utilitarian arguments need further debate and analysis. Like organ procurement during the dying phase of the donor, autonomy and the voluntary nature are fundamental. Although the option of euthanasia through organ donation may be physiologically the best alternative to the DDR and TA-NRP, the question is whether euthanasia through organ donation is ethically more acceptable than TA-NRP as euthanasia is not widely accepted and brings other ethical arguments on the table that need further debate. Euthanasia and donation could strictly be separated by laws and protocols, as is currently the case in cDCD euthanasia (61–63), yet euthanasia through organ donation could blur the line. Furthermore, the public perception may not yet be ready for these approaches. Since maintaining public trust in organ donation is paramount, further explorative research on the perspectives of the public is essential (64). Besides the paradigm shift, abandoning the DDR would also require widespread education of the public to maintain public confidence in the donation process (58, 59).

## Conclusion

In this review we have discussed the current dilemmas regarding TA-NRP for cDCD heart donation and transplantation. There is no consensus on the interpretation of death in a cDCD donor between the countries involved; this finally boils down to the question if we designate the presence of circulation as denominator for life or death, or that more emphasis should be placed on the brain. Furthermore, society and its perspectives on death and organ donation should not be forgotten to ensure trust in voluntary organ donation programs. Prior to further enrollment of TA-NRP, a clear consensus within the medical community should be present regarding the final denominator of circulatory death (i.e., absence of circulation itself vs. absence of brain perfusion), and whether the current available evidence is sufficient to justify its use. At the moment, this seems as one of the most important tasks the transplantation community has to fulfill in the coming years, in order to solidify the role of TA-NRP in cardiac cDCD.

## References

[1] A definition of irreversible coma. Report of the ad hoc committee of the harvard medical school to examine the definition of brain death. JAMA. (1968) 205:337–40. 10.1001/jama.1968.03140320031009 5694976

[2] Guidelines for the determination of death. Report of the medical consultants on the diagnosis of death to the president’s commission for the study of ethical problems in medicine and biomedical and behavioral research. JAMA. (1981) 246:2184–6. 10.1001/jama.1981.03320190042025 7289009

[3] Dhital KK Iyer A Connellan M Chew HC Gao L Doyle A. Adult heart transplantation with distant procurement and ex-vivo preservation of donor hearts after circulatory death: a case series. Lancet. (2015) 385:2585–91. 10.1016/s0140-6736(15)60038-1 25888085

[4] Messer S Page A Axell R Berman M Hernandez-Sanchez J Colah S. Outcome after heart transplantation from donation after circulatory-determined death donors. J Heart Lung Transplant. (2017) 36:1311–8. 10.1016/j.healun.2017.10.021 29173394

[5] Messer S Cernic S Page A Berman M Kaul P Colah S. A 5-year single-center early experience of heart transplantation from donation after circulatory-determined death donors. J Heart Lung Transplant. (2020) 39:1463–75. 10.1016/j.healun.2020.10.001 33248525

[6] Chew HC Iyer A Connellan M Scheuer S Villanueva J Gao L. Outcomes of donation after circulatory death heart transplantation in Australia. J Am Coll Cardiol. (2019) 73:1447–59. 10.1016/j.jacc.2018.12.067 30922476

[7] Scheuer S Chew HC Soto C Iyer A Gao L Hicks M. A single-centre, five-year experience with DCD heart transplantation. Transplantation. (2020) 104:S100. 10.1097/01.tp.0000698768.74526.23

[8] Roest S Kaffka Genaamd Dengler SE van Suylen V van der Kaaij NP Damman K van Laake LW. Waiting list mortality and the potential of donation after circulatory death heart transplantations in The Netherlands.. Neth Heart J. (2021) 29:88–97. 10.1007/s12471-020-01505-y 33156508 PMC7843666

[9] Page A Duehmke R Messer S Barra S Berman M Bhagra S. The impact of DCD heart transplantation on the waiting list: a single centre experience. J Heart Lung Transpant. (2019) 38:S27–8. 10.1016/j.healun.2019.01.051

[10] Holm AM Courtwright A Olland A Zuckermann A Van Raemdonck D. ISHLT Position paper on thoracic organ transplantation in controlled donation after circulatory determination of death (cDCD). J Heart Lung Transplant. (2022) 41:671–7. 10.1016/j.healun.2022.03.005 35370034

[11] Lomero M Gardiner D Coll E Haase-Kromwijk B Procaccio F Immer F. Donation after circulatory death today: an updated overview of the European landscape. Transpl Int. (2020) 33:76–88. 10.1111/tri.13506 31482628

[12] Shemie SD Torrance S Wilson L Hornby L MacLean J Mohr J. Heart donation and transplantation after circulatory determination of death: expert guidance from a Canadian consensus building process. Can J Anaesth. (2021) 68:661–71. 10.1007/s12630-021-01926-2 33543427 PMC8035095

[13] Wall AE Fiedler A Karp S Shah A Testa G. Applying the ethical framework for donation after circulatory death to thoracic normothermic regional perfusion procedures. Am J Transplant. (2022) 22:1311–5. 10.1111/ajt.16959 35040263

[14] Thiessen C Gordon EJ Kelly B Wall A. The ethics of donation after circulatory death organ recovery: an overview of new considerations arising from procurement practice and policy changes. Curr Opin Organ Transplant. (2022) 28:133–8. 10.1097/mot.0000000000001046 36580376

[15] Parent B Caplan A Moazami N Montgomery RA. Response to American college of physician’s statement on the ethics of transplant after normothermic regional perfusion. Am J Transplant. (2022) 22:1307–10. 10.1111/ajt.16947 35072337

[16] Dhital K Ludhani P Scheuer S Connellan M Macdonald Pl. DCD Donations and outcomes of heart transplantation: the Australian experience. Indian J Thorac Cardiovasc Surg. (2020) 36:224–32. 10.1007/s12055-020-00998-x 33061207 PMC7538519

[17] Ethics, determination of death, and organ transplantation in normothermic regional perfusion (NRP) with controlled donation after circulatory determination of death (cDCD): American College of Physicians Statement of Concern. American College of Physicians (2021).

[18] Robertson JA. The dead donor rule. Hastings Center Rep. (1999) 29:6–14. 10.2307/3527865 10641238

[19] Sport MoHWa. Wet op orgaandonatie. In: Ministerie Volksgezondheid WeS, (ed.). *BWBR0008066*. (2020).

[20] Vaststellen van de dood bij postmortale orgaandonatie. The Hague: De Gezondheidsraad (2015). p. 1–160.

[21] Manas D Burnapp L Andrews PA Dark J Mclean A Murphy P Transplantation from donors after deceased circulatory death. British Transplantation Society (2013). 10.1097/01.TP.0000438630.13967.c0 24448588

[22] National protocol for the donation after cardiac death. Organ and Tissue Donation and Transplantation Authority Australia (2010).

[23] Kootstra G Daemen JH Oomen AP. Categories of non-heart-beating donors. Transplant Proc. (1995) 27:2893–4. 7482956

[24] Detry O Le Dinh H Noterdaeme T De Roover A Honoré P Squifflet JP. Categories of donation after cardiocirculatory death. Transplant Proc. (2012) 44:1189–95. 10.1016/j.transproceed.2012.05.001 22663982

[25] Thuong M Ruiz A Evrard P Kuiper M Boffa C Akhtar MZ. New classification of donation after circulatory death donors definitions and terminology. Transpl Int. (2016) 29:749–59. 10.1111/tri.12776 26991858

[26] Bernat JL. The definition and criterion of death. Handb Clin Neurol. (2013) 118:419–35. 10.1016/b978-0-444-53501-6.00033-0 24182395

[27] Shemie SD. Brain arrest, cardiac arrest and uncertainties in defining death. J Pediatr (Rio J). (2007) 83:102–4. 10.2223/jped.1607 17426868

[28] Palleschi A Musso V Mendogni P Zanierato M De Feo TM Cardillo M. Donation after circulatory death program in Italy. Curr Chall Thorac Surg. (2020) 4. 10.21037/ccts-20-116

[29] Dhanani S Hornby L Ward R Shemie S. Variability in the determination of death after cardiac arrest: a review of guidelines and statements. J Intensive Care Med. (2012) 27:238–52. 10.1177/0885066610396993 21841147

[30] Gries CJ White DB Truog RD Dubois J Cosio CC Dhanani S. An official American thoracic society/international society for heart and lung transplantation/society of critical care medicine/association of organ and procurement organizations/united network of organ sharing statement: ethical and policy considerations in organ donation after circulatory determination of death. Am J Respir Crit Care Med. (2013) 188:103–9. 10.1164/rccm.201304-0714ST 23815722

[31] Shemie SD Baker AJ Knoll G Wall W Rocker G Howes D. National recommendations for donation after cardiocirculatory death in Canada: donation after cardiocirculatory death in Canada. CMAJ. (2006) 175:S1. 10.1503/cmaj.060895 17124739 PMC1635157

[32] Longnus SL Mathys V Dornbierer M Dick F Carrel TP Tevaearai HT. Heart transplantation with donation after circulatory determination of death. Nat Rev Cardiol. (2014) 11:354–63. 10.1038/nrcardio.2014.45 24736758

[33] Large S Tsui S Messer S. Clinical and ethical challenges in heart transplantation from donation after circulatory determined death donors. Curr Opin Organ Transplant. (2017) 22:251–9. 10.1097/mot.0000000000000417 28426448

[34] Haase B Bos M Boffa C Lewis P Rudge C Valero R. Ethical, legal, and societal issues and recommendations for controlled and uncontrolled DCD. Transpl Int. (2016) 29:771–9. 10.1111/tri.12720 26581182

[35] Dhanani S Hornby L van Beinum A Scales NB Hogue M Baker A. Resumption of cardiac activity after withdrawal of life-sustaining measures. N Engl J Med. (2021) 384:345–52. 10.1056/NEJMoa2022713 33503343

[36] Tchana-Sato V Ledoux D Detry O Hans G Ancion A D'Orio V. Successful clinical transplantation of hearts donated after circulatory death using normothermic regional perfusion. J Heart Lung Transplant. (2019) 38:593–8. 10.1016/j.healun.2019.02.015 31128600

[37] Anguela-Calvet L Moreno-Gonzalez G Sbraga F Gonzalez-Costello J Tsui S Oliver-Juan E. Heart donation from donors after controlled circulatory death. Transplantation. (2021) 105:1482–91. 10.1097/tp.0000000000003545 33208694

[38] Messer S Page A Colah S Axell R Parizkova B Tsui S. Human heart transplantation from donation after circulatory-determined death donors using normothermic regional perfusion and cold storage. J Heart Lung Transplant. (2018) 37:865–9. 10.1016/j.healun.2018.03.017 29731238

[39] Hoffman JRH McMaster WG Rali AS Rahaman Z Balsara K Absi T. Early US experience with cardiac donation after circulatory death (DCD) using normothermic regional perfusion. J Heart Lung Transplant. (2021) 40:1408–18. 10.1016/j.healun.2021.06.022 34334301

[40] Smith DE Kon ZN Carillo JA Chen S Gidea CG Piper GL. Early experience with donation after circulatory death heart transplantation using normothermic regional perfusion in the United States. J Thorac Cardiovasc Surg. (2022) 164:557–68.e1. 10.1016/j.jtcvs.2021.07.059 34728084

[41] Vandendriessche K Tchana-Sato V Ledoux D Degezelle K Rex S Neyrinck A. Transplantation of donor hearts after circulatory death using normothermic regional perfusion and cold storage preservation. Eur J Cardiothorac Surg. (2021) 60:813–9. 10.1093/ejcts/ezab139 33783513

[42] Pasrija C Tipograf Y Shah AS Trahanas JM. Normothermic regional perfusion for donation after circulatory death donors. Curr Opin Organ Transplant. (2023) 28:71–5. 10.1097/mot.0000000000001038 36409266

[43] Watson CJE Hunt F Messer S Currie I Large S Sutherland A. In situ normothermic perfusion of livers in controlled circulatory death donation may prevent ischemic cholangiopathy and improve graft survival. Am J Transplant. (2019) 19:1745–58. 10.1111/ajt.15241 30589499

[44] Padilla M Coll E Fernández-Pérez C Pont T Ruiz Á Pérez-Redondo M. Improved short-term outcomes of kidney transplants in controlled donation after the circulatory determination of death with the use of normothermic regional perfusion. Am J Transplant. (2021) 21:3618–28. 10.1111/ajt.16622 33891793

[45] De Beule J Vandendriessche K Pengel LHM Bellini MI Dark JH Hessheimer AJ. A systematic review and meta-analyses of regional perfusion in donation after circulatory death solid organ transplantation. Transpl Int. (2021) 34:2046–60. 10.1111/tri.14121 34570380

[46] Alamouti-Fard E Garg P Wadiwala IJ Yazji JH Alomari M Hussain MWA. Normothermic regional perfusion is an emerging cost-effective alternative in donation after circulatory death (DCD) in heart transplantation. Cureus. (2022) 14:e26437. 10.7759/cureus.26437 35800191 PMC9246458

[47] Dalle Ave AL Shaw D Bernat JL. An analysis of heart donation after circulatory determination of death. J Med Ethics. (2016) 42:312–7. 10.1136/medethics-2015-103224 26802005

[48] Bernat JL Capron AM Bleck TP Blosser S Bratton SL Childress JF. The circulatory-respiratory determination of death in organ donation. Crit Care Med. (2010) 38:963–70. 10.1097/CCM.0b013e3181c58916 20124892

[49] Shemie SD. Clarifying the paradigm for the ethics of donation and transplantation: was “dead” really so clear before organ donation? Philos Ethics Humanit Med. (2007) 2(18):18. 10.1186/1747-5341-2-18 17718918 PMC2048971

[50] Manara A Shemie SD Large S Healey A Baker A Badiwala M. Maintaining the permanence principle for death during in situ normothermic regional perfusion for donation after circulatory death organ recovery: a United Kingdom and Canadian proposal. Am J Transplant. (2020) 20:2017–25. 10.1111/ajt.15775 31922653 PMC7540256

[51] Moazami N Weldon E. Heart Transplantation Using Normothermic Regional Perfusion Donation After Circulatory Death. Available at: www.clinicaltrials.gov/ct2/show/NCT04284319

[52] Pana R Hornby L Shemie SD Dhanani S Teitelbaum J. Time to loss of brain function and activity during circulatory arrest. J Crit Care. (2016) 34:77–83. 10.1016/j.jcrc.2016.04.001 27288615

[53] Husain AM. Electroencephalographic assessment of coma. J Clin Neurophysiol. (2006) 23:208–20. 10.1097/01.wnp.0000220094.60482.b5 16751721

[54] Dalsgaard FF Moeslund N Zhang ZL Pedersen M Qerama E Beniczky S. Clamping of the aortic arch vessels during normothermic regional perfusion after circulatory death prevents the return of brain activity in a porcine model. Transplantation. (2022) 106:1763–9. 10.1097/tp.0000000000004047 35066546

[55] Ribeiro R Alvarez J Yu F Gomes B Hondjeu A Adamson M. Assessment of cerebral perfusion and activity during normothermic regional perfusion in a porcine model of donation after circulatory death. J Heart Lung Transpant. (2021) 40:S234. 10.1016/j.healun.2021.01.673

[56] Khalil K Ribeiro RVP Alvarez JS Badiwala MV Der Sarkissian S Noiseux N. Large-animal model of donation after circulatory death and normothermic regional perfusion for cardiac assessment. J Vis Exp. (2022) 183:e64009. 10.3791/64009 35635480

[57] Perez-Villares JM Rubio JJ Del Río F Miñambres E. Validation of a new proposal to avoid donor resuscitation in controlled donation after circulatory death with normothermic regional perfusion. Resuscitation. (2017) 117:46–9. 10.1016/j.resuscitation.2017.05.030 28591558

[58] Evans DW. Seeking an ethical and legal way of procuring transplantable organs from the dying without further attempts to redefine human death. Philos Ethics Humanit Med. (2007) 2:11. 10.1186/1747-5341-2-11 17603889 PMC1920527

[59] Verheijde JL Rady MY McGregor J. Recovery of transplantable organs after cardiac or circulatory death: transforming the paradigm for the ethics of organ donation. Philos Ethics Humanit Med. (2007) 2:8. 10.1186/1747-5341-2-8 17519030 PMC1892566

[60] Truog RD Robinson WM. Role of brain death and the dead-donor rule in the ethics of organ transplantation. Crit Care Med. (2003) 31:2391–6. 10.1097/01.Ccm.0000090869.19410.3c 14501972

[61] Mulder H Olthuis G Siebelink M Gerritsen R van Heurn E. [Guideline “organ donation following euthanasia”]. Ned Tijdschr Geneeskd. (2017) 161:D2135. 29219798

[62] Downar J Shemie SD Gillrie C Fortin MC Appleby A Buchman DZ. Deceased organ and tissue donation after medical assistance in dying and other conscious and competent donors: guidance for policy. CMAJ. (2019) 191:E604–13. 10.1503/cmaj.181648 31160497 PMC6546574

[63] Mulder J Sonneveld H Van Raemdonck D Downar J Wiebe K Domínguez-Gil B. Practice and challenges for organ donation after medical assistance in dying: a scoping review including the results of the first international roundtable in 2021. Am J Transplant. (2022) 22:2759–80. 10.1111/ajt.17198 36100362 PMC10092544

[64] Bollen JAM Shaw D de Wert G Ten Hoopen R Ysebaert D van Heurn E. Euthanasia through living organ donation: ethical, legal, and medical challenges. J Heart Lung Transplant. (2019) 38:111–3. 10.1016/j.healun.2018.07.014 30197210

